# The ecological effects of selective decontamination of the digestive tract (SDD) on antimicrobial resistance: a 21-year longitudinal single-centre study

**DOI:** 10.1186/s13054-019-2480-z

**Published:** 2019-06-07

**Authors:** Sophie Buitinck, Rogier Jansen, Saskia Rijkenberg, Jos P. J. Wester, Rob J. Bosman, Nardo J. M. van der Meer, Peter H. J. van der Voort

**Affiliations:** 1 Department of Intensive Care, OLVG Hospital, Oosterpark 9, 1091 AC Amsterdam, The Netherlands; 2 TIAS School for Business and Society, Warandelaan 2, 5037 AB Tilburg, The Netherlands; 3 Department of Medical Microbiology, OLVG Hospital, Oosterpark 9, 1091 AC Amsterdam, The Netherlands; 4 Department of Intensive Care, Amphia Hospital, Molengracht 21, 4814 CK Breda, The Netherlands

**Keywords:** Selective digestive tract decontamination, SDD, Resistance, Ecological effect, Critically ill

## Abstract

**Background:**

The long-term ecological effects on the emergence of antimicrobial resistance at the ICU level during selective decontamination of the digestive tract (SDD) are unknown. We determined the incidence of newly acquired antimicrobial resistance of aerobic gram-negative potentially pathogenic bacteria (AGNB) during SDD.

**Methods:**

In a single-centre observational cohort study over a 21-year period, all consecutive patients, treated with or without SDD, admitted to the ICU were included. The antibiotic regime was unchanged over the study period. Incidence rates for ICU-acquired AGNB’s resistance for third-generation cephalosporins, colistin/polymyxin B, tobramycin/gentamicin or ciprofloxacin were calculated per year. Changes over time were tested by negative binomial regression in a generalized linear model.

**Results:**

Eighty-six percent of 14,015 patients were treated with SDD. Most cultures were taken from the digestive tract (41.9%) and sputum (21.1%). A total of 20,593 isolates of AGNB were identified. The two most often found bacteria were *Escherichia coli* (*N* = 6409) and *Pseudomonas* (*N* = 5269). The incidence rate per 1000 patient-day for ICU-acquired resistance to cephalosporins was 2.03, for polymyxin B/colistin 0.51, for tobramycin 2.59 and for ciprofloxacin 2.2. The incidence rates for ICU-acquired resistant microbes per year ranged from 0 to 4.94 per 1000 patient-days, and no significant time-trend in incidence rates were found for any of the antimicrobials. The background prevalence rates of resistant strains measured on admission for cephalosporins, polymyxin B/colistin and ciprofloxacin rose over time with 7.9%, 3.5% and 8.0% respectively.

**Conclusions:**

During more than 21-year SDD, the incidence rates of resistant microbes at the ICU level did not significantly increase over time but the background resistance rates increased. An overall ecological effect of prolonged application of SDD by counting resistant microorganisms in the ICU was not shown in a country with relatively low rates of resistant microorganisms.

**Electronic supplementary material:**

The online version of this article (10.1186/s13054-019-2480-z) contains supplementary material, which is available to authorized users.

## Background

Selective decontamination of the digestive tract (SDD) is effective in preventing intensive care unit (ICU)-acquired pneumonia, sepsis and subsequent mortality [1–4]. This is due to the elimination of potentially pathogenic microbes from the digestive tract using topical antibiotics [5]. As a consequence, secondary colonization and subsequent endogenous infection are prevented [5]. This routine use of antimicrobials raises concerns for antimicrobial resistance, and these concerns are the most important reason to oppose the use of SDD [6]. Some reports, however, show that the elimination of pathogens from the bowel might even reduce the emergence of resistant strains [7–10]. Two meta-analyses on the effect of selective oral decontamination (SOD) and SDD on the development of antimicrobial resistance showed no relation between the use of SDD or SOD and the emergence of antimicrobial resistance [11, 12]. Most included studies were randomized controlled trials that focussed on individual patients treated with SDD but they seldomly included the patients in the ICU without SDD. As a consequence, at the ICU level, in contrast to the individual patient level, the effect on microbial resistance could not be determined with these studies. Moreover, these studies generally reported a relatively short inclusion period.

The aim of the present study is to determine the incidence of antimicrobial resistance in aerobic gram-negative potentially pathogenic microorganisms (AGNB) to the components of SDD and other frequently used antibiotics at ICU level over a 21-year period with an unchanged antibiotic policy.

## Methods

### Study design, setting and patients

This study has a single-centre longitudinal observational cohort design. The patient data are registered in a local database since 1997 and retrospectively extracted for this analysis. The study is conducted in a Dutch 20-bed adult mixed medical, surgical and cardiac surgery tertiary intensive care unit. All consecutive patients admitted to the ICU between January 1997 and July 2017 were included, and clinical data were extracted. All available cultures and patient data were used. Patients without cultures taken during ICU treatment could not be counted in the culture data but are included in the other analyses. Data on baseline characteristics of all included patients were prospectively recorded in the ICU database and extracted for this analysis.

The local medical ethical review board (ACWO OLVG) approved the study and waived informed consent due to its retrospective design in accordance with Dutch and European legislation (study no. WO 13.063).

### Cultures

Data on all cultures taken during ICU stay of the included patients were over the years registered in the hospital database and retrospectively retrieved from this database. Both surveillance cultures (throat, rectum, tracheal aspirate) and cultures of organ sites and blood taken during ICU stay were included for analysis. Routinely, cultures from the throat and rectum were taken twice a week and tracheal aspirate three times a week for surveillance in patients treated with SDD. For non-intubated patients, the tracheal aspirate was substituted for sputum when available. All culture samples taken in the context of surveillance were plated on an unselective blood agar and four specific agars selecting for gram-positive bacteria, gram-negative bacteria, yeast and vancomycin-resistant enterococci. Gram-negatives were cultured on McConkey agar; *Staphylococcus aureus* was detected using both unselective blood agar and Columbia aztreonam polymyxin agar. For yeasts, Sabouraud maltose agar was used. Cultured gram-negative bacteria were tested for antimicrobial susceptibility using agar disk diffusion during the entire study period. Cutoff values for resistance were set following the guidelines of the ‘Clinical and Laboratory Standards Institute’ (CLSI) [13] until July 2011 and ‘the European Committee on Antimicrobial Susceptibility Testing’ (EUCAST) from July 2011 until 2017 [14]. Cutoff values for resistance for colistin were performed according to CLSI ranges, for the whole study period.

### Antimicrobial treatment 1997–2017

Patients were treated with SDD when the expected ICU stay was more than 24 h, with or without mechanical ventilation. This relatively liberal use of SDD is based on the concept that acquisition of potential pathogenic microorganisms starts in the first day of ICU stay [15, 16]. SDD has been consistently used since 1986 following the same protocol. The SDD regimen consists of four times daily Orabase® oral paste with 2% polymyxin B, amphotericin B and tobramycin. In addition, 10 ml of a suspension containing 500 mg amphotericin B, 100 mg polymyxin B and 80 mg tobramycin is administered four times daily in the gastric tube or swallowed in patients without a gastric tube. Cefotaxime is administered four times daily 1 g i.v. for 4 days in all SDD-treated patients but is administered longer in case of active infection. For active infection, the coverage of gram-negatives can be extended with ciprofloxacin i.v. or tobramycin i.v. when needed. In case of peritonitis, metronidazole is added as well. Other i.v. antimicrobials can be given based on previous culture results. Penicillins are carefully avoided whenever possible due to their negative effects on the normal aerobic and anaerobic intestinal flora which could lead to a loss of the protective effect to invading pathogens (colonization resistance). Methicillin-resistant staphylococci (MRSA) colonization and infection were consistently treated with vancomycin i.v. and 2% vancomycin added in the oral paste and gastric suspension. When a combined polymyxin and tobramycin resistance was present in AGNB from surveillance cultures, then co-trimoxazole 2% was added in the oral paste and twice daily 960 mg in the enteral suspension when appropriate and until decontamination was obtained [15].

### Data analysis and statistics

Cultures with aerobic gram-negative potentially pathogenic bacteria (AGNB) were selected. Among others, these AGNB are A*cinetobacter* spp., *Citrobacter* spp*., Escheria coli*, *Enterobacter* spp., *Klebsiella* spp., *Morganella* spp., *Proteus* spp., *Pseudomonas aeruginosa*, and *Serratia marcescens* [16]. To obtain the resistant strains for analysis, we selected in every patient the AGNB with antimicrobial resistance for tobramycin/gentamicin, polymyxin B/colistin, cephalosporins or ciprofloxacin and counted the first culture with this particular resistance. The strains were defined as resistant when they were tested intermediate or resistant in vitro. Strains that were resistant on admission were marked as not ICU-acquired and were excluded for the calculation of the incidence rates but they were analyzed separately to calculate the background resistance rates on admission. Resistant pathogens were considered ICU-acquired if these pathogens were absent in the first 48 to 72 h after admission but present in subsequent cultures. In case a patient enters the ICU with resistant bacteria and subsequently acquires other resistant bacteria, they are counted in both groups. Because of variability in time of admission and time that the culture was taken, the cultures taken on day three are considered within 48 to 72 h after admission. As a consequence, all resistant pathogens from cultures taken on day of admission (day one), day two or day three were considered resistant on admission and not ICU acquired. This is consistent with the definition of ICU-acquired infection in ‘the ECDC HAIICU protocol for healthcare associated infections in intensive care units’ [17].

### Prevalence rate and incidence rate

The incidence rates for ICU-acquired resistance were calculated for each year for second- or third-generation cephalosporins (including cefotaxime, ceftriaxone, cefuroxim and ceftazidim), polymyxin B/colistin, tobramycin/gentamicin and ciprofloxacin separately. Microorganisms that were intrinsically resistant to one of the antimicrobial agents were excluded for the analysis of that specific agent. For third-generation cephalosporins, we excluded *Serratia marcescens*, *Acinetobacter* spp., *Citrobacter* freundii, *Enterobacter* spp. and *Morganella* morgannii, and for polymyxinB/colistin, *Serratia marcescens*, *Proteus* spp. and *Morganella* spp. were excluded for this calculation. Incidence rates were calculated instead of cumulative incidence because of the dynamic population [18]. The incidence rate was defined as:

[Number of ICU-acquired resistant PPMs/sum of the person-time of the population at risk] × 1000.

Population at risk was defined as all patients admitted to the ICU during the study period without resistant strains on admission. Patients who were treated less than 48 h in the ICU were excluded from the denominator as they could not have acquired new (multi)resistant strains in this short period. The person-time, calculated in patient-days per year, per patient at risk is defined as the number of days between ICU admission and discharge from the ICU. The number of days was calculated without correcting for the time of admission or discharge. The day of admission is ‘day one’ and so forth. In case of acquisition of a resistant potentially pathogenic microorganism (PPM), person-time was defined as the number of patient-days between ICU admission and acquisition of this resistant PPM. The total number of patient-days for patients at risk was calculated for every antimicrobial agent separately. Incidence rates were calculated for patients with an ICU length of stay more than 48 h and for all patients (Additional files 1, 2, 3 and 4), for medical and surgical patients (Additional files 1, 2, 3 and 4).

### Statistical analyses

Continuous data are shown as mean and standard deviation (SD) or median and interquartile range (IQR) depending on the distribution. Changes in incidence over time were evaluated by negative binomial regression in a generalized linear model, as the assumption for Poisson distribution that mean and variance are equal was not met (overdispersion). The dependent variable is the incidence rate per 1000 patient-days of ICU-acquired resistant strains for a given antimicrobial agent over the years of measurement. The Omnibus test was used to test for significance. It compares the model including the variable ‘year’ to the intercept-only model to see if there is a significant time-trend. A two-sided *p* value below 0·05 was considered statistically significant.

To determine background resistance rates, the prevalence of resistance on admission in AGNB was calculated for each year separately. The prevalence was calculated by dividing the number of resistant strains on admission by the number of admissions in that particular year. All data were analyzed using Statistical Package of Social Science (SPSS 24.0 Chicago, IL, USA).

## Results

### Patients and cultures

Between January 1, 1997, and July 29, 2017, 15,051 patients were admitted to the ICU. These patients had 17,511 admissions with cultures taken during ICU stay. Baseline characteristics are shown in Table 1. In the second column, all patients with an ICU length of stay less than 48 h are excluded as they could not acquire new colonization with resistant strains in this short period. Over time, the case mix did not change substantially with around half of the population having cardiovascular disease and the other mixed diseases including sepsis and abdominal surgery. The distribution of sample sites is also shown in Table 1. The majority of the cultures were surveillance cultures from the throat or rectum (41.9%) or cultures retrieved from tracheal aspirate (23.3%).

**Table 1:** Baseline characteristics

		All patients	More than 48 h ICU stay
Patients and cultures baseline characteristics			
Number of admissions between 1997 and 2017		17,511	9588
Age in years, mean (SD)		65.3 (14.5)	66.0 (13.6)
Males, *N* (%)/females *N* (%)		11,093 (63.4%)/6414 (36.6%)	6077 (63.4%)/3508 (36.3%)
Length of ICU stay in days, median (IQR)		3.0 (4.0)	6.0 (6.0)
APACHE II score	Median (IQR)	20.0 (11.0)	22.0 (11.0)
	APACHE II < 20, *N* (%)	8281 (47.3%)	3428 (35.8%)
	APACHE II > = 20, *N* (%)	8320 (47.5%)	5767 (60.1%)
	Missing, *N* (%)	910 (5.6%)	393 (4.1%)
Patient category	Cardiothoracic surgery, *N* (%)	6109 (34.9%)	3021 (31.5%)
	Internal medicine, *N* (%)	2434 (13.9%)	1666 (17.4%)
	Surgery, *N* (%)	3361 (19.2%)	1751 (18.2%)
	Cardiology, *N* (%)	2468 (14.1%)	1540 (16.1%)
	Pulmonology, *N* (%)	1529 (8.7%)	1047 (10.9%)
	Neurology, *N* (%)	527 (3.0%)	299 (3.1%)
	Other, *N* (%)	1009 (5.8%)	264 (2.8%)
	Missing, *N* (%)	74 (0.4%)	3 (0.03%)
Admission type	Medical*, *N* (%)	9335 (53.3%)	5787 (60.4%)
	Elective surgery, *N* (%)	3964 (22.6%)	1696 (17.7%)
	Scheduled surgery, *N* (%)	1264 (7.2%)	532 (5.5%)
	Urgent surgery, *N* (%)	1553 (8.9%)	825 (8.6%)
	Emergency surgery, *N* (%)	624 (3.6%)	358 (3.7%)
	Missing, *N* (%)	771 (4.4%)	390 (4.1%)
Treated with SDD^#^	Yes, *N* (%)	12,054 (86.0%)	7141 (93.5%)
	No, *N* (%)	1043 (7.4%)	83 (1.1%)
	Missing, *N* (%)	919 (6.6%)	416 (5.4%)
ICU mortality, *N* (%)		2459 (14.0%)	1537 (16.0%)
Cultures			
Distribution of specimen	Digestive tract cultures, *N* (%)	68,057 (41.9%)	
	Sputum cultures, *N* (%)	37,827 (23.3%)	
	Skin and wound cultures	7308 (4.5%)	
	Urine cultures	8212 (5.1%)	
	Blood cultures	27,884 (17.2%)	
	Abdominal cavity cultures	1125 (0.7%)	
	Other	11,236 (6.9%)	
	Unknown	586 (0.4%)	
	Total number of cultures	162,235	

Continuous data are shown as mean and standard deviation (SD) in case of normal distribution

*Medical patients are all patients without previous surgery preceding ICU admission

^#^Data available since 2002

**Fig. 1:**
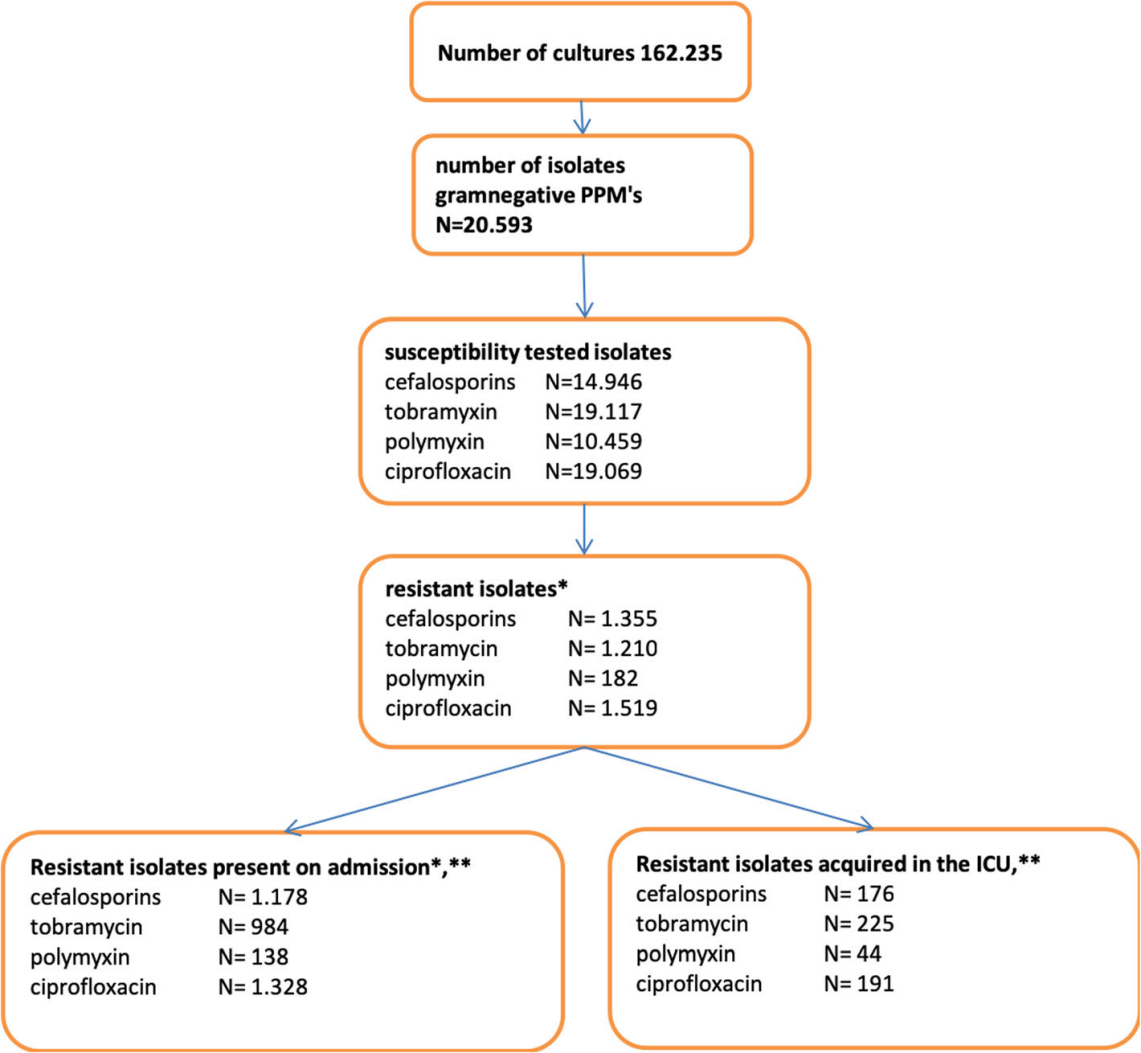
Flowchart of cultures. *Only first resistant isolate mentioned in flowchart. **First culture with antimicrobial resistance taken within 72 h after admission

Figure 1 shows the number of cultures and number of resistant strains. A total of 20,593 isolates of AGNB were identified. These included *Proteus* spp. (*N* = 2857), *Klebsiella* spp*.* (*N* = 1634), *Morganella* spp. (*N* = 759), *Enterobacter* spp*.* (*N* = 1571), *Citrobacter* spp. (*N* = 529), *Serratia* marcescens (*N* = 1081), *Pseudomonas* spp. (*N* = 5269), *Acinetobacter* spp. (*N* = 485) and *E. coli* (*N* = 6409).

For the second- or third-generation cephalosporins, the number of isolates that were tested for resistance increased from 78.4% in 1997 to 99.2% in 2017. A higher test rate leads to a better detection of resistant microorganisms. Susceptibility for ciprofloxacin was tested for 78.4% in 1997 and 99.2% in 2017. In 65.8% of isolates, information on susceptibility to polymyxin B/colistin was available. The number of tested isolates for polymyxin B/colistin rose from 22.7% in 1997 to 99.0% in 2017. Information on susceptibility for aminoglycosides was available in 92.9% of all isolates. This number varied between 78.8 and 99.2% over the years. More information on susceptibility testing per year for the different antimicrobial agents is shown in Additional files 1, 2, 3 and 4.

**Fig. 2:**
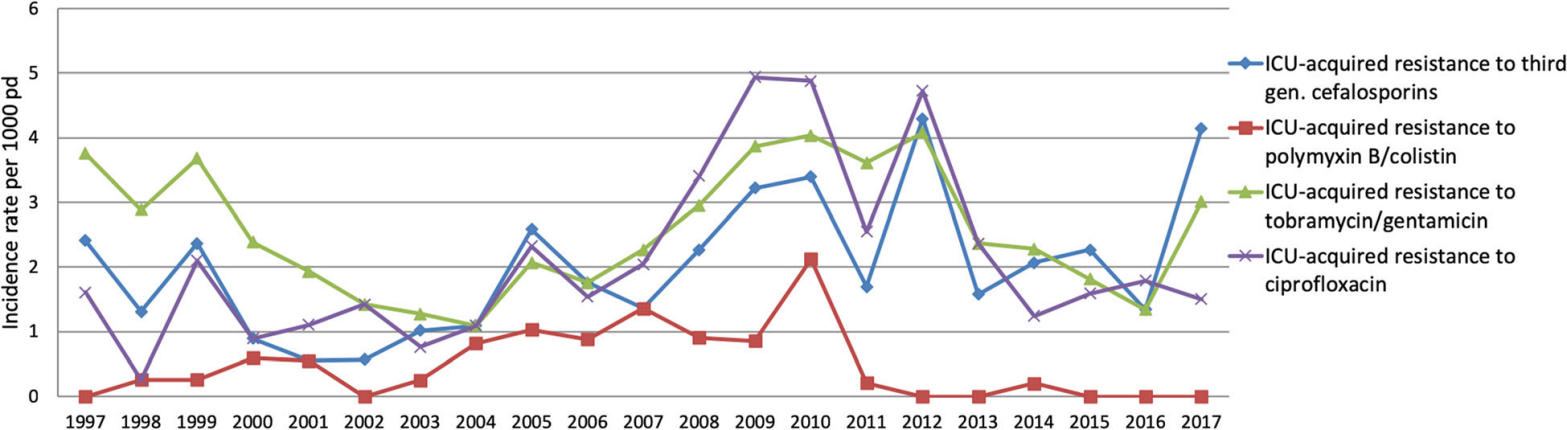
Incidence rates per 1000 patient-days for acquired resistance

The incidence rate per 1000 patient-days per year is shown in Fig. 2. These data reflect the population of ICU patients with a length of stay more than 48 h. In Additional files 1, 2, 3 and 4, the data including the patients with a shorter length of stay are shown. The average incidence rate for ICU-acquired resistance to third-generation cephalosporins was 2.03 per 1000 patient-days (range 0.55/1000 patient-days in 2001 to 4.3/1000 patient-days in 2012), 0.51 per 1000 patient-days for polymyxin B/colistin (range 0.00/1000 patient-days in 1997 to 2.12/1000 patient-days in 2010), 2.59 per 1000 patient-days for tobramycin (range 1.09/1000 in 2004 to 4.08/1000 patient-days in 2012) and 2.20 per 1000 patient-days for ciprofloxacin (range 0.26/1000 patient-days in 1998 to 4.94 per 1000 patient-days in 2009. The results of the negative binomial regression analysis are listed in Table 2.

**Table 2:** Incidence of acquired antimicrobial resistance over time

Antimicrobial agent	All patients	LOS > 48 h	Surgical	Medical
Third generation cephalosporins	0.063 (*p* = 0.998)	0.071 (*p* = 0.79)	0.14 (*p* = 0.71)	0.009 (*p* = 0.92)
Tobramycin/gentamicin	0.39 (*p* = 0.999)	0.09 (*p* = 0.76)	0.070 (p = 0.79)	0.10 (*p* = 0.75)
Ciprofloxacin	0.88 (*p* = 0.985)	0.04 (*p* = 0.84)	0.034 (*p* = 0.85)	0.30 (*p* = 0.58)
Polymyxin B/Colistin	0.07 (*p* = 0.899)	0.26 (*p* = 0.61)	0.54 (*p* = 0.46)	0.46 (*p* = 0.50)

Data are shown as likelihood ratio chi-square test (Omnibus test) to compare the fitted model (model including year) against the intercept-only model with *p* value

*LOS* length of stay in the ICU

The incidence rates for medical and surgical patients have been calculated separately. Additional files 3 and 4 Medical and Surgical show the incidence rates over time for these two groups. Basically, it is shown that in both groups the incidence rates are low with some variability over the years.

**Fig. 3:**
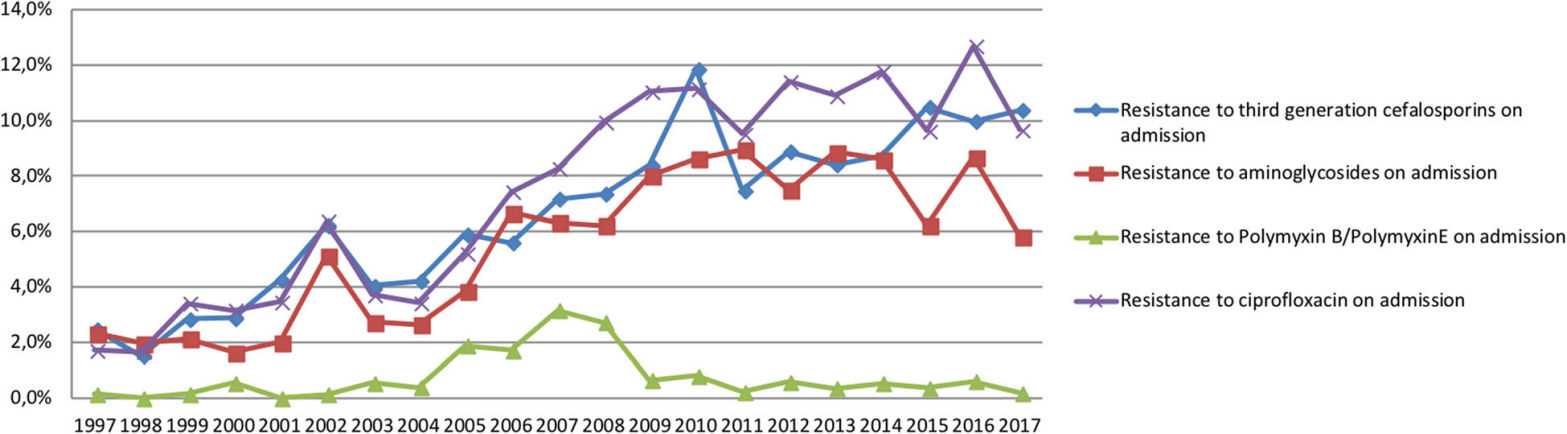
Admission prevalence rates for resistant antimicrobials per year

The prevalence rates of resistant aerobic gram-negative PPMs on admission per year are presented in Fig. 3, and this represents the ‘background’ prevalence. Overall prevalence rate of resistance to cephalosporins was 6.7 per 100 ICU admissions. This was 0.8/100 admissions for polymyxin B/colistin, 5.6/100 admissions for tobramycin and 7.6/100 admissions for ciprofloxacin. Figure 3 shows an increase in background resistance for cephalosporins, ciprofloxacin and tobramycin but for polymyxin B/colistin this trend is not seen.

## Discussion

This study shows that some fluctuation in the incidence of newly acquired resistant microorganisms over 21 years of SDD exists but a significant or relevant increase in resistance over the study period was not found despite the fact that the number of patients with one or more resistant strains on admission was steadily increasing. The long-term ecological effects of SDD on ICU level concerning antimicrobial resistance of aerobic gram-negative PPMs to the components of SDD appear to be limited. The incidence rates for ICU-acquired bacteria resistant for third-generation cephalosporins, tobramycin/gentamicin, polymyxin B/colistin and ciprofloxacin appeared to be very low in this study, between 0 and 6 per 1000 patient-days for each antimicrobial agent. We analyzed the data excluding and including patients with a length of stay less than 48 h and for medical and surgical patients separately. Basically, all analyses showed the same result. These findings are consistent with most of the previous studies on the emergence of antimicrobial resistance during SDD use [10–12, 19–23]. Halaby et al., in contrast, showed an increased prevalence (the incidence was not reported) of colistin-resistant microorganisms which probably was due to failure of hygienic measures in that ICU [24]. In addition, Oostdijk et al. reported a trend to an increased incidence of aminoglycosides [25]. Moreover, several studies showed a decrease in resistance after implementation of SDD [10, 19–21].

This study is the first to report uninterrupted use of SDD over a time period of 21 years. As SDD started in 1985 in this ICU, in fact, more than 30 years of uninterrupted use did not lead to emergence of resistance. We can only report 21 years as the electronic registration of culture results started in 1994. Studies on the long-term effects of the emergence of resistant microorganism and studies at the ICU level of SDD are scarce [12]. The second strength of this study is that patients without SDD treatment have been included as well. As a result, a more comprehensive overview of the effects of SDD can be obtained at the unit level. Some of these effects might have stayed unrecognized when in a RCT design, only patients that are included in the study would be evaluated leaving others, potentially carrying resistant strains, unnoticed. Other reservoirs in the ICU than patients, such as sinks or equipment, were not checked for the presence of resistant microorganisms in this study. In the context of SDD, these reservoirs should be dealt with by hygienic measures, one of the pillars of the SDD regime.

Little changes have been made in the antibiotic policy of our ICU in the past 20 years. In particular, SDD has been used continuously, consistently and unchanged in composition during the whole 21-year study period. The exposure to the antimicrobials is therefore long enough to unravel ecological trends.

This study also has limitations. First, the longitudinal cohort analysis of this study has its methodological flaws. It cannot compare groups with or without SDD but that was not the primary aim of this study. The primary aim was to study the ecological effects on unit level for which a randomized study is less appropriate. As causal effects are difficult to determine, we can only conclude that long-term SDD use was not associated with increasing incidence of resistant strains. In addition, we did not take rectal and throat cultures from patients who were not treated with SDD which leaves us unknown about their colonization. It is unknown whether these patients introduce resistant microorganism in the ICU. Also, they might take acquired multiresistant microorganisms with them out of the ICU but the exposure (colonization pressure) appears to be low. Second, because of its mono-centred design, the extrapolation to other ICUs is difficult to make. More ICUs with the same length of SDD use were not available, which limited the analysis to a single centre. Third, in this study, we limited our resistance rates to second/third-generation cephalosporins, tobramycin/gentamicin, polymyxin B/colistin and ciprofloxacin in aerobic gram-negative PPMs. We did not study other antimicrobials or gram-positive microorganisms as previous studies did not find an emergence of resistant gram-positive bacteria like enterococci [26, 27].

Fourth, the guidelines for susceptibility testing have been changed several times between 1997 and 2017, which could lead to differences in classification at different time periods. In our centre, the guidelines have been changed from CLSI to EUCAST guidelines in 2011. As EUCAST is known to recommend lower resistance MIC breakpoints, it can hypothetically lead to higher resistance rates. However, our results did not show this. This is confirmed by several studies that have been published on the effect of changing guidelines on resistance rates [28, 29]. When comparing CLSI 2009 guidelines and EUCAST 2010 guidelines, the effects of changing guidelines were small and would have led to higher instead of lower resistance rates [28, 29].

Fifth, detailed information on a patient level concerning the use of antibiotics in our ICU was not available for most of the study period. Also, the time window between admission and the detection of newly acquired antimicrobial resistance is unknown but would have been informative in relation to the length of stay. Sixth, we have studied this cohort in a country with a relatively low prevalence of resistant bacteria. However, we have also shown that the increasing background prevalence did not influence the incidence of newly acquired resistant strains in the ICU. This stresses the importance of hygienic measures as one of the pillars of SDD treatment [16]. In Fig. 2, a temporary increase in ICU-acquired resistance between 2007 and 2012 was seen. The subsequent decrease might be related to a policy change resulting in more patients in preventive isolation on admission, which might have reduced cross contamination from newly admitted patients to others. It may also indicate that transmission is a more important mechanism than new formation or selection of resistant strains.

We have included both patients with and without mechanical ventilation, which is not in all ICUs and studies routine. Over the years, around 85% of our population is mechanically ventilated during their stay in the ICU but we do not have exact data available for this study. It is unknown whether this ‘liberal’ SDD policy relates to the incidence rates that we report here. In addition, the median length of stay of all patients is 3.0 days, which is relatively short for newly acquired resistant microorganisms. Therefore, we analyzed the patients with a length of stay of more than 48 h who appeared to have a median length of stay of 6.0 days (Table 1).

This study is performed in a setting with a low baseline prevalence of resistant microorganisms. A rise in background rates of resistant microorganisms is present, also in the Netherlands. Despite this rise (Fig. 3), watchful application of SDD and adaptation of the substances that are used when necessary did not lead to an increase in the incidence of resistant microorganisms. In a recent study, Wittekamp et al. showed no effect of SDD on bacteraemia in a setting of high prevalence of resistant microorganisms. The limitation of that study is that they did not adapt the composition of the SDD substances to the specific patterns of resistant microorganisms as we did in our unit [30].

## Conclusions

This study shows that long-term use of SDD can be safe in relation to the emergence of resistant microbes as our study found that between 1997 and 2017, the incidence of ICU-acquired antimicrobial resistance to second/third-generation cephalosporins, tobramycin/gentamicin, polymyxin B/colistin and ciprofloxacin in AGNB was low and did not increase over time with uninterrupted use of SDD. This is seen despite increased background resistance rates on admission due to an overall increase of antimicrobial resistance in the hospital population in a country with on average low rates of resistant microorganisms.

## Additional files

Additional file 1: Percentages of cultures with susceptibility testing. (PDF 150 kb)

Additional file 2: Incidence rates all patients. (TIFF 4487 kb)

Additional file 3: Incidence rates medical patients. (TIFF 4487 kb)

Additional file 4: Incidence rates surgical patients. (TIFF 4572 kb)

## Data Availability

Data are available in the electronic patient records. Anonymous datasets are generated and stored in a secured place in the hospital’s electronic system for medical research.

## Abbreviations

AGNB: Aerobic gram-negative potentially pathogenic bacteria
CLSI: Clinical and Laboratory Standards Institute
ECDC: European Centre for Disease Prevention and Control
EUCAST: European Committee on Antimicrobial Susceptibility Testing
HAIICU: Healthcare-associated infections in intensive care units
ICU: Intensive care unit
IQR: Interquartile range
MEC: Medical Ethical review board
MRSA: Methicillin-resistant *Staphylococcus aureus*
PPM: Potentially pathogenic microorganism
RCT: Randomized controlled trial
SD: Standard deviation
SDD: Selective digestive tract decontamination
SOD: Selective oral decontamination
SPSS: Statistical Package for the Social Science
