# Supplementary material for: The ecological effects of selective decontamination of the digestive tract (SDD) on antimicrobial resistance: a 21-year longitudinal single-centre study
Source: Crit Care. 2019 Jun 7;23:208. doi: 10.1186/s13054-019-2480-z (PMC6555978; doi:10.1186/s13054-019-2480-z)
Supplement: Supplementary file 1 — Percentages of cultures with susceptibility testing. (PDF 150 kb) [file 13054_2019_2480_MOESM1_ESM.pdf]

	3rd gen Cefalosporins			Polymyxine B/Colestin			Aminoglycosides			Ciprofloxacin		
	number of isolates	isolates tested		number of isolates	isolates tested		number of isolates	isolates tested		number of isolates	isolates tested	
1997	541	424	78,4%	517	112	21,7%	685	540	78,8%	685	537	78,4%
1998	542	451	83,2%	504	104	20,6%	734	592	80,7%	734	590	80,4%
1999	610	508	83,3%	578	199	34,4%	779	657	84,3%	779	652	83,7%
2000	494	420	85,0%	437	152	34,8%	605	516	85,3%	605	515	85,1%
2001	613	506	82,5%	654	265	40,5%	833	689	82,7%	833	687	82,5%
2002	843	766	90,9%	809	385	47,6%	1094	1003	91,7%	1094	1002	91,6%
2003	708	643	90,8%	634	358	56,5%	867	791	91,2%	867	789	91,0%
2004	784	718	91,6%	713	407	57,1%	951	879	92,4%	951	877	92,2%
2005	843	769	91,2%	815	454	55,7%	1057	976	92,3%	1057	977	92,4%
2006	915	845	92,3%	919	514	55,9%	1236	1131	91,5%	1236	1130	91,4%
2007	1002	920	91,8%	978	444	45,4%	1254	1155	92,1%	1254	1154	92,0%
2008	911	868	95,3%	885	431	48,7%	1191	1133	95,1%	1191	1133	95,1%
2009	976	915	93,8%	962	542	56,3%	1246	1177	94,5%	1246	1178	94,5%
2010	1020	977	95,8%	1017	718	70,6%	1226	1176	95,9%	1226	1176	95,9%
2011	853	812	95,2%	876	853	97,4%	1075	1052	97,9%	1075	1051	97,8%
2012	919	903	98,3%	972	959	98,7%	1173	1156	98,6%	1173	1154	98,4%
2013	789	757	95,9%	850	826	97,2%	1067	1040	97,5%	1067	1040	97,5%
2014	852	822	96,5%	810	798	98,5%	1056	1025	97,1%	1056	1025	97,1%
2015	688	673	97,8%	705	690	97,9%	897	879	98,0%	897	879	98,0%
2016	832	824	99,0%	849	844	99,4%	1050	1041	99,1%	1050	1041	99,1%
2017	429	425	99,1%	408	404	99,0%	513	509	99,2%	513	509	99,2%
total	16164	14946	**92,5%**	15892	10459	**65,8%**	20589	19117	**92,9%**	20589	19096	**92,7%**
