# Supplementary figures and images for: The ecological effects of selective decontamination of the digestive tract (SDD) on antimicrobial resistance: a 21-year longitudinal single-centre study
Source: Crit Care. 2019 Jun 7;23:208. doi: 10.1186/s13054-019-2480-z (PMC6555978; doi:10.1186/s13054-019-2480-z)

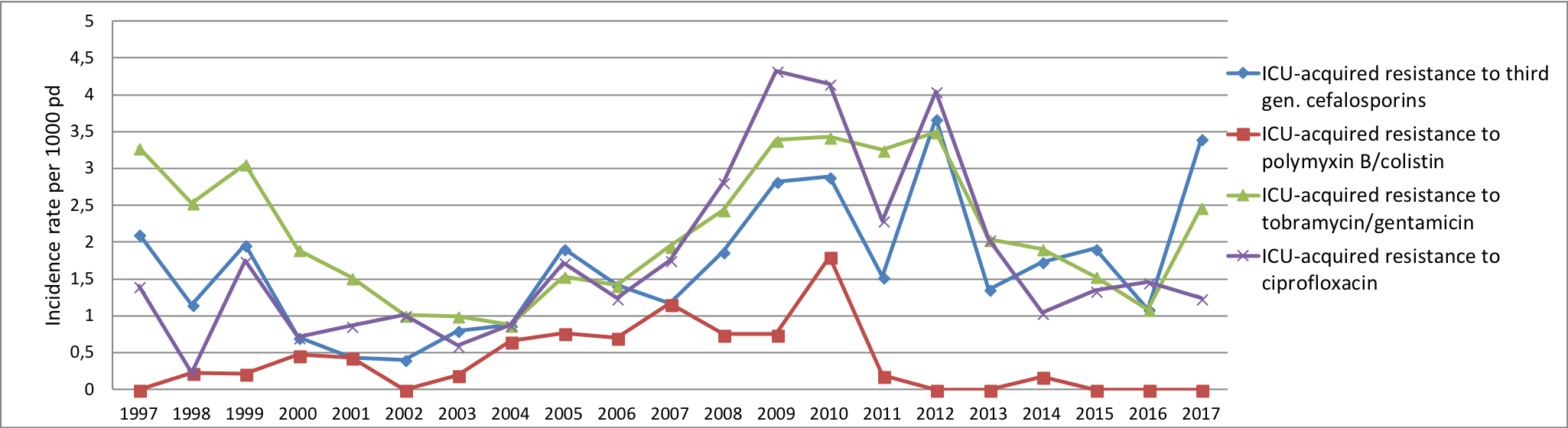

Supplement: Supplementary file 2 — Incidence rates all patients. (TIFF 4487 kb) [file 13054_2019_2480_MOESM2_ESM.tiff]

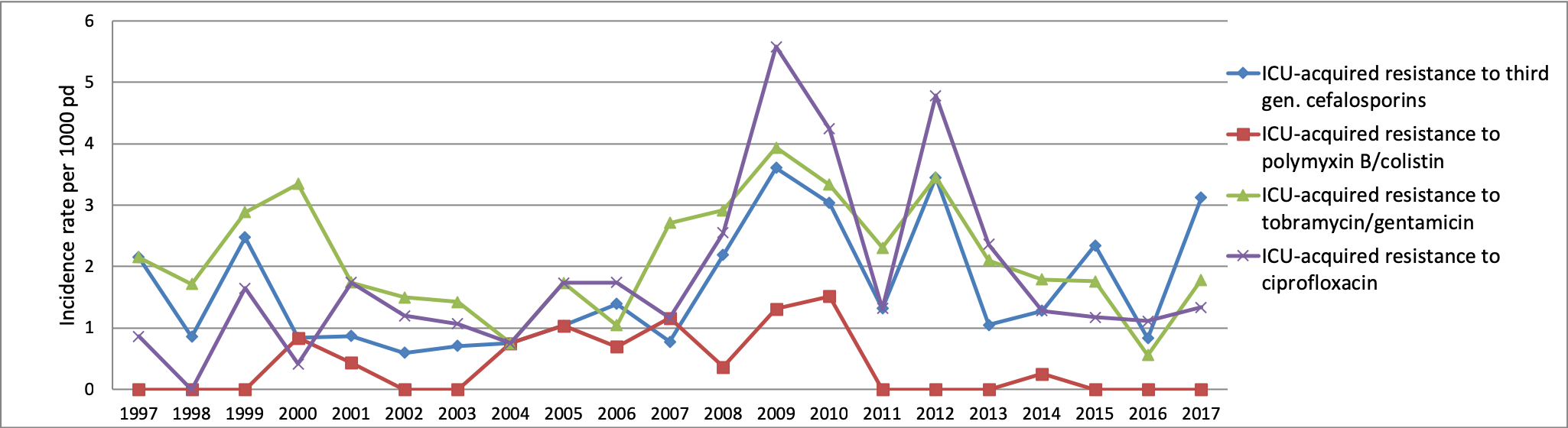

Supplement: Supplementary file 3 — Incidence rates medical patients. (TIFF 4487 kb) [file 13054_2019_2480_MOESM3_ESM.tiff]

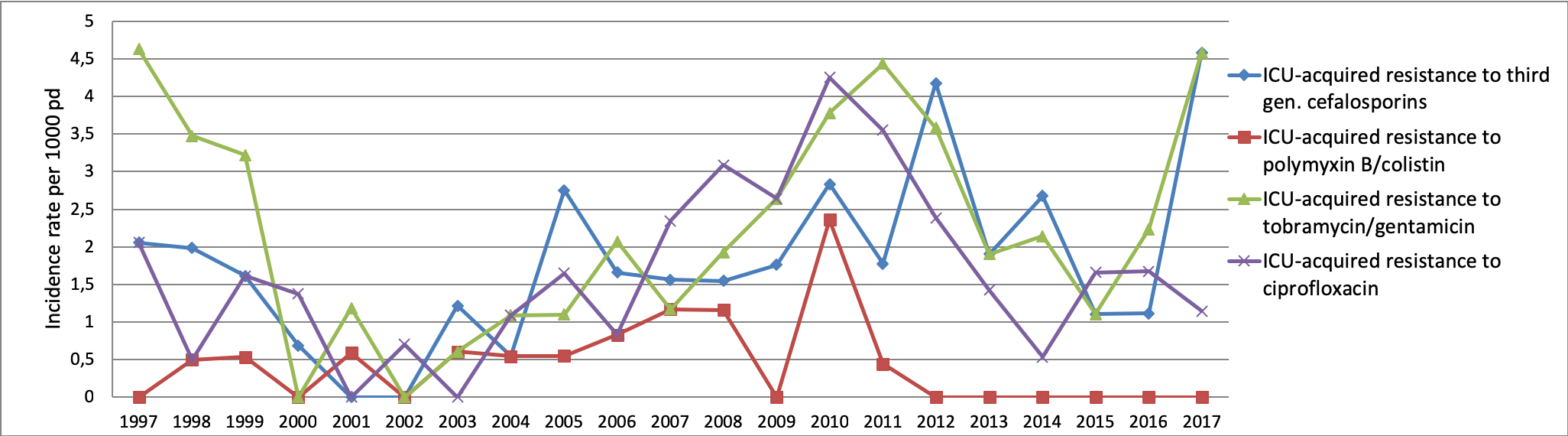

Supplement: Supplementary file 4 — Incidence rates surgical patients. (TIFF 4572 kb) [file 13054_2019_2480_MOESM4_ESM.tiff]
